# Supplementary material for: Hot spot prediction in protein-protein interactions by an ensemble system
Source: BMC Syst Biol. 2018 Dec 31;12(Suppl 9):132. doi: 10.1186/s12918-018-0665-8 (PMC6311905; doi:10.1186/s12918-018-0665-8)
Supplement: Supplementary file 2 — All relevant MATLAB code. A simple Matlab implement of our predictor. (ZIP 19,742 kb) [file 12918_2018_665_MOESM2_ESM.zip › Additional file 2/Read ASA/ASA website/dbMPIKT/4KROB.html]

 NetSurfP - Protein Surface Accessibility and Secondary Structure Predictions

  

|  |  |  |
| --- | --- | --- |
|  |  | NetSurfP - Protein Surface Accessibility and Secondary Structure Predictions  Technical University of Denmark |

  


---

```
# For publication of results, please cite:
# A generic method for assignment of reliability scores applied to solvent accessibility predictions.
# Bent Petersen, Thomas Nordahl Petersen, Pernille Andersen, Morten Nielsen and Claus Lundegaard
# BMC Structural Biology 2009, 9:51 doi:10.1186/1472-6807-9-51
#
# Column 1: Class assignment - B for buried or E for Exposed - Threshold: 25% exposure, but not based on RSA
# Column 2: Amino acid
# Column 3: Sequence name
# Column 4: Amino acid number
# Column 5: Relative Surface Accessibility - RSA
# Column 6: Absolute Surface Accessibility
# Column 7: Z-fit score for RSA prediction
# Column 8: Probability for Alpha-Helix
# Column 9: Probability for Beta-strand
# Column 10: Probability for Coil
E Q  4KROB                  1    0.797 142.273   1.326   0.003   0.003   0.994
B V  4KROB                  2    0.187  28.696  -0.363   0.004   0.514   0.481
E Q  4KROB                  3    0.391  69.833   1.252   0.003   0.718   0.279
B L  4KROB                  4    0.142  25.945   0.718   0.018   0.846   0.136
E Q  4KROB                  5    0.363  64.814   0.679   0.021   0.756   0.223
B E  4KROB                  6    0.224  39.115   0.525   0.022   0.552   0.426
E S  4KROB                  7    0.379  44.384  -0.477   0.020   0.205   0.775
E G  4KROB                  8    0.441  34.699  -1.764   0.018   0.088   0.893
E G  4KROB                  9    0.505  39.736  -2.256   0.018   0.047   0.935
E G  4KROB                 10    0.489  38.453  -1.676   0.019   0.141   0.840
E L  4KROB                 11    0.356  65.275  -0.600   0.022   0.359   0.619
E V  4KROB                 12    0.305  46.848   0.566   0.021   0.451   0.528
E Q  4KROB                 13    0.389  69.529   1.045   0.020   0.205   0.775
E P  4KROB                 14    0.468  66.395  -0.657   0.018   0.047   0.935
E G  4KROB                 15    0.595  46.858  -2.149   0.005   0.015   0.979
E G  4KROB                 16    0.436  34.305  -0.867   0.004   0.085   0.910
E S  4KROB                 17    0.390  45.743  -0.691   0.005   0.336   0.660
B L  4KROB                 18    0.157  28.765   0.719   0.003   0.718   0.279
E R  4KROB                 19    0.387  88.715   0.768   0.001   0.900   0.099
B L  4KROB                 20    0.064  11.792   0.615   0.001   0.900   0.099
B S  4KROB                 21    0.131  15.412   0.693   0.001   0.900   0.099
B C  4KROB                 22    0.035   4.914   0.056   0.001   0.900   0.099
B A  4KROB                 23    0.264  29.104   0.635   0.001   0.900   0.099
B A  4KROB                 24    0.080   8.783  -0.420   0.003   0.718   0.279
B S  4KROB                 25    0.168  19.690  -0.576   0.004   0.420   0.576
B G  4KROB                 26    0.219  17.220  -1.820   0.004   0.138   0.858
B R  4KROB                 27    0.243  55.716  -0.833   0.005   0.262   0.733
B T  4KROB                 28    0.147  20.403  -1.006   0.004   0.420   0.576
B F  4KROB                 29    0.109  21.796  -1.162   0.021   0.451   0.528
B S  4KROB                 30    0.186  21.811  -1.306   0.021   0.279   0.699
B S  4KROB                 31    0.277  32.511  -1.300   0.018   0.088   0.893
B Y  4KROB                 32    0.099  21.135   0.156   0.019   0.141   0.840
B A  4KROB                 33    0.070   7.758   0.177   0.021   0.451   0.528
B M  4KROB                 34    0.023   4.662   0.691   0.021   0.756   0.223
B G  4KROB                 35    0.047   3.730  -0.058   0.018   0.846   0.136
B W  4KROB                 36    0.032   7.816   0.523   0.018   0.846   0.136
B F  4KROB                 37    0.035   7.004   0.291   0.018   0.846   0.136
B R  4KROB                 38    0.107  24.526   0.532   0.023   0.655   0.322
B Q  4KROB                 39    0.087  15.538   0.211   0.022   0.359   0.619
B A  4KROB                 40    0.191  21.092   0.695   0.005   0.045   0.951
E P  4KROB                 41    0.333  47.210   0.617   0.005   0.015   0.979
E G  4KROB                 42    0.530  41.727  -1.537   0.018   0.019   0.964
E K  4KROB                 43    0.409  84.214   0.578   0.018   0.019   0.964
E Q  4KROB                 44    0.427  76.262  -0.392   0.018   0.088   0.893
B R  4KROB                 45    0.158  36.159   0.817   0.005   0.336   0.660
B E  4KROB                 46    0.104  18.204  -0.208   0.003   0.718   0.279
B F  4KROB                 47    0.046   9.333   0.233   0.001   0.900   0.099
B V  4KROB                 48    0.024   3.643   0.980   0.001   0.959   0.040
B A  4KROB                 49    0.017   1.906   0.042   0.001   0.959   0.040
B A  4KROB                 50    0.077   8.441  -0.744   0.001   0.959   0.040
B I  4KROB                 51    0.021   3.867   1.091   0.001   0.959   0.040
B R  4KROB                 52    0.137  31.419   0.760   0.002   0.816   0.182
B W  4KROB                 53    0.266  63.973  -0.974   0.005   0.336   0.660
E S  4KROB                 54    0.532  62.397  -1.634   0.005   0.015   0.979
E G  4KROB                 55    0.526  41.365  -2.509   0.018   0.019   0.964
E G  4KROB                 56    0.545  42.899  -2.090   0.018   0.019   0.964
E Y  4KROB                 57    0.480 102.619  -0.615   0.018   0.088   0.893
E T  4KROB                 58    0.308  42.747   0.211   0.020   0.205   0.775
E Y  4KROB                 59    0.412  87.938   0.226   0.021   0.279   0.699
B Y  4KROB                 60    0.189  40.389   0.575   0.064   0.216   0.721
B T  4KROB                 61    0.199  27.643  -0.335   0.052   0.084   0.864
E D  4KROB                 62    0.623  89.760  -0.543   0.053   0.043   0.903
E S  4KROB                 63    0.381  44.688   0.067   0.052   0.084   0.864
B V  4KROB                 64    0.078  11.989  -0.486   0.020   0.205   0.775
E K  4KROB                 65    0.377  77.569   0.836   0.020   0.205   0.775
B G  4KROB                 66    0.209  16.440  -1.523   0.005   0.336   0.660
B R  4KROB                 67    0.222  50.792   0.888   0.003   0.718   0.279
B F  4KROB                 68    0.050  10.075  -0.151   0.001   0.900   0.099
B T  4KROB                 69    0.134  18.627   0.921   0.001   0.900   0.099
B I  4KROB                 70    0.044   8.177   0.912   0.001   0.900   0.099
B S  4KROB                 71    0.106  12.400   0.493   0.018   0.846   0.136
B R  4KROB                 72    0.244  55.807   0.789   0.022   0.552   0.426
B D  4KROB                 73    0.241  34.714  -0.324   0.022   0.359   0.619
E N  4KROB                 74    0.430  62.923   0.179   0.018   0.088   0.893
B A  4KROB                 75    0.226  24.872  -1.799   0.018   0.047   0.935
E K  4KROB                 76    0.524 107.766   1.065   0.056   0.142   0.802
E T  4KROB                 77    0.264  36.589  -0.224   0.021   0.279   0.699
B T  4KROB                 78    0.166  23.080   0.843   0.021   0.756   0.223
B V  4KROB                 79    0.070  10.790  -0.836   0.018   0.846   0.136
B Y  4KROB                 80    0.165  35.325   0.414   0.011   0.918   0.071
B L  4KROB                 81    0.046   8.514   0.632   0.018   0.846   0.136
B Q  4KROB                 82    0.135  24.111  -0.295   0.021   0.756   0.223
B M  4KROB                 83    0.083  16.528  -0.621   0.021   0.451   0.528
E N  4KROB                 84    0.338  49.512  -0.369   0.020   0.205   0.775
E S  4KROB                 85    0.373  43.774  -0.625   0.018   0.088   0.893
B L  4KROB                 86    0.087  15.985   0.068   0.018   0.047   0.935
E K  4KROB                 87    0.467  96.124   1.197   0.018   0.047   0.935
E P  4KROB                 88    0.362  51.311  -0.606   0.053   0.043   0.903
E E  4KROB                 89    0.435  76.012  -0.616   0.053   0.043   0.903
B D  4KROB                 90    0.158  22.768  -0.964   0.053   0.043   0.903
B T  4KROB                 91    0.105  14.577   0.301   0.135   0.317   0.548
B A  4KROB                 92    0.036   3.956  -0.394   0.087   0.683   0.230
B V  4KROB                 93    0.067  10.344   0.962   0.085   0.775   0.140
B Y  4KROB                 94    0.036   7.608   0.892   0.072   0.855   0.073
B Y  4KROB                 95    0.049  10.450   0.955   0.085   0.775   0.140
B C  4KROB                 96    0.016   2.204   1.529   0.087   0.683   0.230
B A  4KROB                 97    0.029   3.152   0.135   0.022   0.552   0.426
B A  4KROB                 98    0.127  14.039   0.209   0.021   0.279   0.699
E T  4KROB                 99    0.307  42.636  -0.344   0.019   0.141   0.840
B Y  4KROB                100    0.292  62.443  -0.715   0.018   0.088   0.893
B L  4KROB                101    0.322  59.050  -1.710   0.018   0.088   0.893
E S  4KROB                102    0.417  48.849  -1.952   0.018   0.088   0.893
E S  4KROB                103    0.385  45.110  -2.380   0.018   0.088   0.893
E D  4KROB                104    0.360  51.919  -2.498   0.018   0.088   0.893
E Y  4KROB                105    0.377  80.608  -2.111   0.018   0.088   0.893
E S  4KROB                106    0.422  49.482  -2.279   0.018   0.088   0.893
E R  4KROB                107    0.360  82.371  -2.111   0.018   0.088   0.893
E Y  4KROB                108    0.371  79.197  -2.136   0.018   0.088   0.893
E A  4KROB                109    0.351  38.636  -2.268   0.018   0.088   0.893
E L  4KROB                110    0.329  60.167  -2.286   0.018   0.088   0.893
E P  4KROB                111    0.340  48.203  -2.071   0.018   0.047   0.935
B Q  4KROB                112    0.281  50.115  -1.671   0.018   0.047   0.935
E R  4KROB                113    0.367  83.951  -1.361   0.018   0.047   0.935
E P  4KROB                114    0.370  52.460  -1.660   0.018   0.047   0.935
E L  4KROB                115    0.317  58.024  -1.241   0.018   0.047   0.935
B D  4KROB                116    0.164  23.690  -1.132   0.018   0.088   0.893
B Y  4KROB                117    0.063  13.420  -0.101   0.019   0.141   0.840
B D  4KROB                118    0.072  10.346  -0.463   0.020   0.205   0.775
B Y  4KROB                119    0.059  12.651  -0.539   0.020   0.205   0.775
B W  4KROB                120    0.043  10.414  -0.448   0.019   0.141   0.840
B G  4KROB                121    0.035   2.739  -1.841   0.019   0.141   0.840
B Q  4KROB                122    0.127  22.772  -0.086   0.018   0.088   0.893
B G  4KROB                123    0.094   7.374  -1.213   0.005   0.262   0.733
B T  4KROB                124    0.061   8.461   0.090   0.004   0.514   0.481
E Q  4KROB                125    0.223  39.881   0.138   0.018   0.846   0.136
B V  4KROB                126    0.038   5.764   0.509   0.011   0.918   0.071
B T  4KROB                127    0.088  12.150   0.605   0.018   0.846   0.136
B V  4KROB                128    0.070  10.728   0.008   0.021   0.756   0.223
B S  4KROB                129    0.200  23.405  -0.529   0.004   0.420   0.576
B S  4KROB                130    0.237  27.800  -0.814   0.020   0.205   0.775
E L  4KROB                131    0.377  69.029  -1.594   0.018   0.047   0.935
E E  4KROB                132    0.435  76.064   0.292   0.018   0.047   0.935
E H  4KROB                133    0.434  78.999  -0.813   0.018   0.047   0.935
E H  4KROB                134    0.588 107.048  -0.488   0.018   0.047   0.935
E H  4KROB                135    0.370  67.339  -1.734   0.018   0.047   0.935
E H  4KROB                136    0.387  70.359  -0.915   0.018   0.047   0.935
E H  4KROB                137    0.579 105.229  -0.574   0.018   0.047   0.935
E H  4KROB                138    0.808 146.957  -1.056   0.003   0.003   0.994

Explain the output.  Go back.
```

---
